# Epidemiology of epizootic lymphangitis of carthorses in northern Ethiopia using conventional diagnostic methods and nested polymerase chain reaction

**DOI:** 10.1186/s12917-020-02582-2

**Published:** 2020-10-07

**Authors:** Birhanu Hadush, Molla Michaelay, Habtamu Taddele Menghistu, Nigus Abebe, Abreha Tesfaye Genzebu, Habtom Kiros Bitsue, Berihun Afera, Bojia E. Duguma, Getachew Gugsa, Gobena Ameni

**Affiliations:** 1 College of Veterinary Sciences, Mekelle University, P.O. Box 2084, Mekelle, Ethiopia; 2 Department of Animal Sciences, Raya University, P.O. Box 42, Maichew, Ethiopia; 3 Donkey Sanctuary of Ethiopia, P.O.Box 1055, 1250 Africa Avenue Road, Addis Ababa, Bole Sub City, Ethiopia; 4 School of Veterinary Sciences, Wollo University, P.O.Box 1145, Dessie, Ethiopia; 5 Aklilu Lema Institute of Pathobiology, Addis Ababa University, P.O. Box 1176, Addis Ababa, Ethiopia

**Keywords:** Epizootic lymphangitis, Gram stain, Histoplasma capsulatum var farciminosum, PCR

## Abstract

**Background:**

Epizootic lymphangitis (EL), caused by *Histoplasma capsulatum variety farciminosum* (HCF) is a contagious, chronic disease of equines, characterized by development of nodular lesions in the lymph nodes, lymphatic vessels and skin. It is one of the most important diseases of equines in Ethiopia, causing significant economic loss, particularly in the livelihood of carthorse owners. To date there is neither effective diagnostic nor control measure implemented in the country. Furthermore, there is a shortage of data on the epidemiology of the disease in different regions of this country. The aim of this study was to investigate epidemiology of EL in northern Ethiopia, using the conventional methods as well as nested polymerase chain reaction (PCR).

**Results:**

The presence of HCF genetic material was confirmed in 44% (84/191) of the carthorses. Subclinical infection was observed in 18.2% (22/121) of the apparently healthy carthorses. Considering the nested PCR as a gold standard, sensitivity and specificity of clinical examination were 74% and 92.5%, respectively, while the area under the ROC curve (AUR) was 0.83 (95% confidence interval, 0.77, 0.896). Moreover, a moderate (*k* = 0.675) agreement observed between the nested PCR and clinical examination.

**Conclusions:**

This study demonstrated widespread occurrence of EL in northern Ethiopia, and the advantage of the nested PCR in detecting infection of HCF, even before the clinical symptoms became apparent.

## Background

Ethiopia is home to 8.85 million donkeys, 2.01 million horses, and 0.46 million mules. With 2.01 million head of horses, Ethiopia accounts for about 34.5% of the total African equine population, and 3.45% of the global population [1]. In Ethiopia, equines are used for transportation of people and commodities, as well as in support of crop production. These equids are exposed to both husbandry mismanagement and diseases caused by bacteria, viruses, fungi and parasites. Epizootic lymphangitis (EL), also called Equine histoplasmosis (EH), has been a priority disease of high morbidity and mortality with significant economic impact [2].

It is caused by *Histoplasma (H.) capsulatum var. faciminosum* (HCF); a dimorphic fungus that exists in soil as a mold and transforms into yeast forms once it parasitizes mammalian tissues. Most infections in humans are ascribed to *Histoplasma (H.) capsulatum var. capsulatum (*HCC), while HCF is an equine pathogen [3]. Epizootic lymphangitis is a contagious and chronic disease of horses and other Equidae. Clinically it is characterized by a spreading, suppurative, ulcerating pyogranulomatous dermatitis and lymphangitis [4].

The disease is endemic in sub Saharan Africa and especially in Ethiopia, but previous reports indicate the presence of the disease within European, Northern African and Asian countries [5–7]. However, knowledge on the current global epidemiological situation of the disease is limited.

A gross examination study conducted in selected towns indicated that the disease is endemic in Ethiopia with a prevalence ranging from 0–39.1% [2, 4, 8, 9]. Epizootic lymphangitis has a significant economic impact on the livelihood of poor carthorse owners where a morbidity of a horse could result in more than a 50% reduction in daily earnings. The effect of this disease on resource poor families, as well as animal welfare can be devastating. This could be exacerbated by ineffective, costly or unavailable treatment of the animal, resulting in abandoning of severely infected horses [10–14]. Successful EL control measures are non-existent in Ethiopia, therefore it remains the number one priority disease for horses, which affects the socio-economic and welfare of communities who are dependent on carthorse as a means of livelihood.

Currently, diagnosis of EL in Ethiopia is based on clinical symptoms and microscopic examination for HCF yeast cells within pus. Though, such classical diagnostic methods are useful for routine case management in endemic areas, they are not suitable for detection of asymptomatic carriers due to limited specificity and sensitivity. Thus, evaluation of PCR based diagnostic protocols to rapidly identify HCF directly from equine clinical specimens is of paramount importance to understand the molecular epidemiology of the disease [15]. The aim of this study was to investigate the epidemiology of EL in northern Ethiopia using the conventional methods in combination with PCR and to evaluate the inherent diagnostic capacity of conventional tests (clinical sign and microscopic examination) for diagnosis of EL in carthorses.

## Results

### Description of study animals and clinical observations

**Fig. 1:**
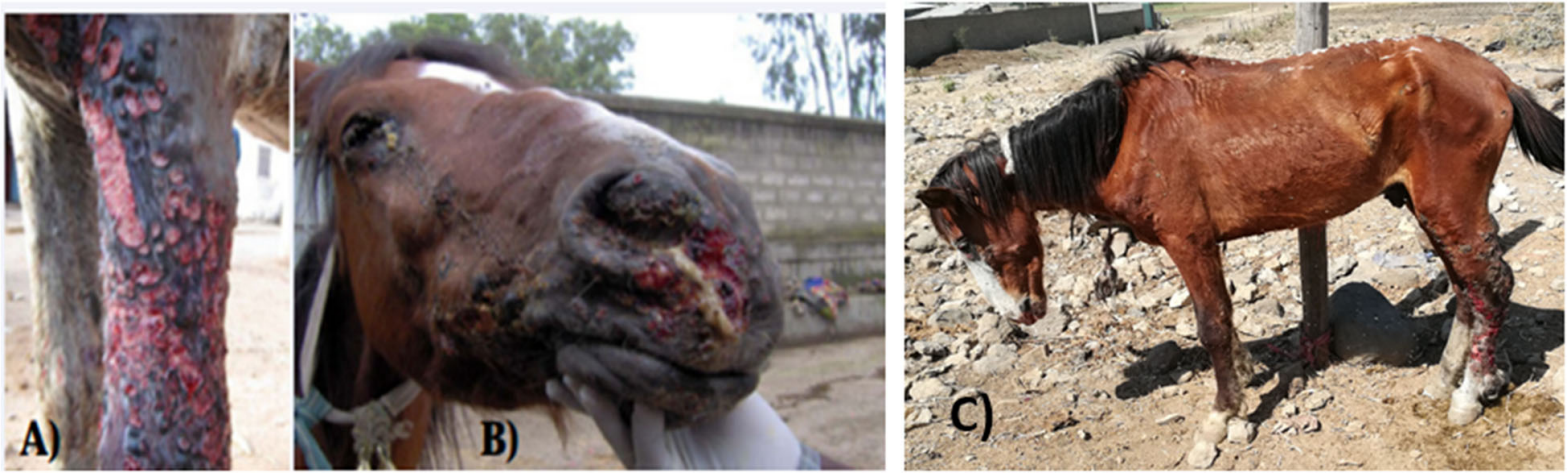
Clinical forms of epizootic lymphangitis. **a**. cutaneous form in fore limb, **b**. pulmonary form and **c**. cutaneous form in hind limb

Carthorses included in this study were from Mekelle (*n* = 61; 31.9%), Bati (*n* = 36; 18.8%), Kamisse (*n* = 42; 22%) and Kombolcha (*n* = 52; 27.2%). Two age categories were defined either as 3–6 years (*n* = 39; 20.4%) or above 6 years (*n* = 152; 79.6%) (Table 1). Body conditions of the carthorses were poor (*n* = 09; 4.7%), moderate (*n* = 30; 15.7%), and good (*n* = 152; 79.6%). Out of 191 carthorses observed, 70 showed clinical signs of EL (Fig. 1). Thirty-two (*n* = 32; 16.7%) out of the 191 carthorses had more than two un-ruptured nodules.

**Table 1:** PCR results and their association with age, body condition, study area and clinical condition

Variables		PCR result		***X***^***2***^	df	***p*** value
		pos	neg			
Age (years)	3-6	5	34	32.31	2	<0.001
	above 6	79	73			
Body condition	poor	7	2	21.59	2	<0.001
	moderate	23	7			
	good	54	98			
City	Mekelle	35	26	3.07	3	0.381
	Bati	16	20			
	Kamisse	18	24			
	Kombolcha	15	37			
Clinical condition	with suspected EL lesion	62	8	89.18	1	<0.001
	apparently healthy	22	99			

*pos* positive, *neg* negative

### Gram stain and culture

**Fig. 2:**
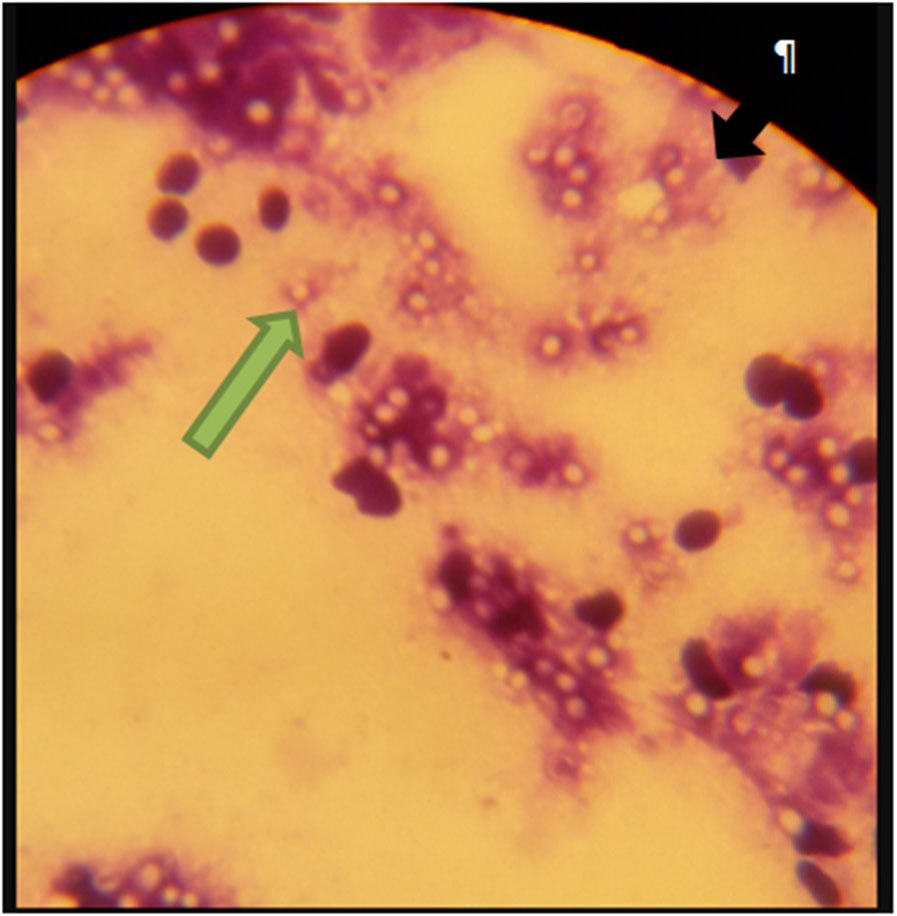
Gram stained smears of pus samples from Closed EL nodules. Green arrow=. Individual *Histoplasma casulatum var farciminosum (HCF)* yeast cell; black arrow= HCF yeast cells intracellularly within the macrophages

On gram stain and microscopy, 93.75% (30/32) of the samples showed positive microscopic features of the yeast phase of HCF, characterized by Gram-positive yeast forms that are round to oval with one edge wider and the other bluntly pointed in shape with a halo (unstained capsule-like) structure or with faint blue cytoplasmic space. They were occurring individually or in groups, either free (Fig. 2, green arrow) or intracellularly within the macrophages (Fig. 2, black arrow). Only one yeast form of HCF was isolated.

### PCR

**Fig. 3:**
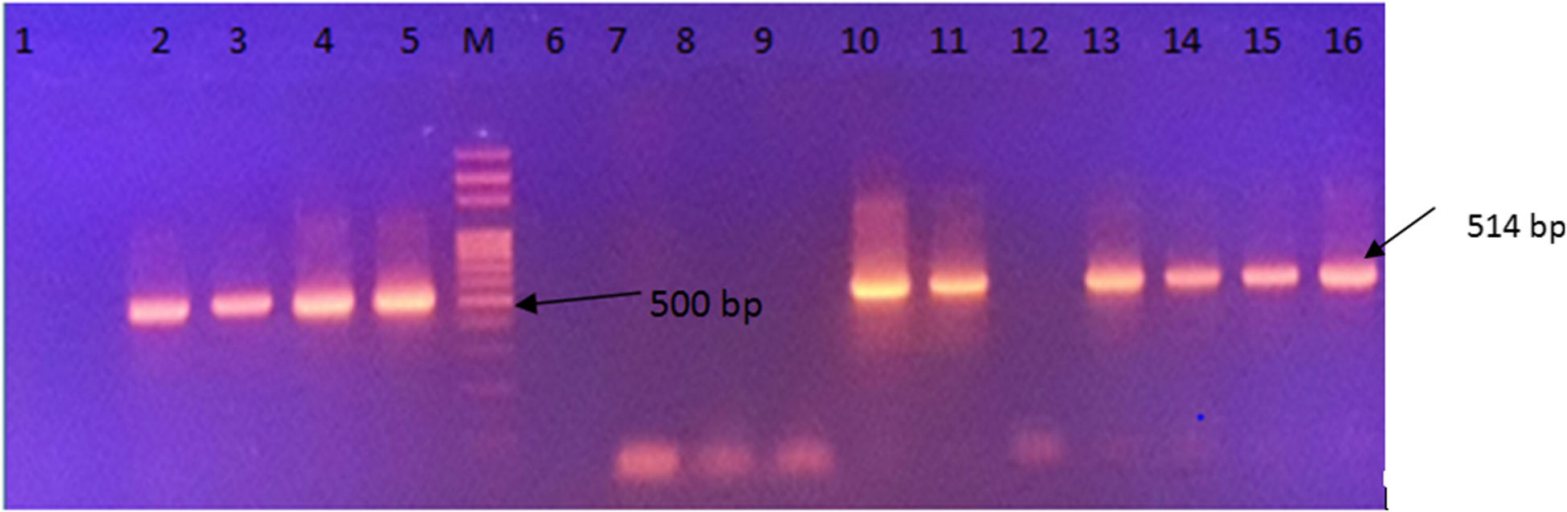
Gel electrophoresis of nested PCR amplification products obtained from DNA extracted from horse buffy coat samples. Lane 1: negative control, lanes 2-5 buffy coat DNAs of apparently healthy horses that gave positive amplicon, M= marker, lanes 6-9 buffy coat DNAs of apparently healthy horses with no amplification, lanes 10-15 buffy coat DNAs of clinically suspected EL cases giving positive amplification except lane 12, and lane 16 positive control

The presence of HCF genetic material using the nested PCR was detected in 44% (84/191) of the carthorses, (Tables 1 and 2). Of the 70 carthorses with clinical suspicions of EL, 88.6% (62/70) were PCR positive while of the 121 apparently healthy carthorses, 18.2% (22/121) were PCR positive. The size of the PCR amplicons was 514 bp (Fig. 3).

**Table 2:** Odds ratio for risk factors associated with the occurrence EL

Variables		COR	95% CI	*p* value	B	AOR	95% CI	*p* value	B
Age (years)	3–6	1				1			
	above 6	7.359	2.73– 19.83	< 0.001	1.996	9.660	3.21–29.11	< 0.001	2.269
Body condition	Poor	1				1			
	Moderate	0.939	0.16– 5.59	0.945	-0.06	0.770	0.10–5.68	0.797	-0.262
	Good	0.157	0.03– 0.79	0.024	-1.84	0.105	0.02–0.66	0.016	-2.253
Clinical condition	apparently healthy	1				1			
	with EL lesion	3.552	14.62–83.18	< 0.001	3.552	30.309	11.17–82.23	< 0.001	3.411

*COR* Crude Odds Ratio, *CI *Confidence Interval, *B *intercept and *AOR* Adjusted Odds Ratio

There was very strong evidence of a relationship between age and being positive for EL using PCR (*X*^2^ = 32.31, df = 2, *p* < 0.001). The odds ratio of being positive for HCF using PCR for carthorses above 6 years was 9.66 times higher than horses with ages of 3–6 years (AOR = 9.66, 95% CI: 3.206–29.109, *p* < 0.001). Moreover, there was a statistically significant association between body condition score and detection of HCF nucleic acid (*X*^2^ = 21.59, df = 2, *p* < 0.001) (Table 1). Carthorses with moderate and good body condition were 0.77 (AOR = 0.77, 95% CI: 0.104–5.681, p = 0.797) and 0.105 (AOR = 0.105, 95% CI: 0.017–0.658, *p* = 0.016) times less likely to be positive for HCF infection, respectively, than those that have poor body condition.

There was no evidence of relationship between the study area and being positive for HCF using PCR (*X*^2^ = 3.1, df = 3, *p* = 0.381). The odds ratio of being positive for HCF using PCR for carthorses with EL lesions was 30.309 times higher than apparently healthy horses (AOR = 30.309, 95% CI: 11.2–82.2, *p* < 0.001) (Table 2).

### Diagnostic accuracy of clinical observation and gram stain

Taking PCR as a gold standard/reference test, 191 buffy coat samples and 32 pus smears/gram stains of clinically suspected animals were represented in a contingency Table (Table 3). The sensitivity and specificity of clinical signs respectively was 73.81% and 92.52% while the sensitivity and specificity of gram stain was 99.67% and 50%, respectively. The test agreement between clinical observation and PCR was good (*k* = 0.675, 95% CI 0.570–0.78), while the test agreement between gram stain and PCR test was moderate (*k* = 0.467, 95% CI -0.160–1.000).

**Table 3:** Contingency table with results of all buffy coat samples tested in the PCR, Clinical signs and gram stain; and diagnostic accuracy of clinical observation with PCR

	**Type of diagnostic tests**			
	**Clinical observation(*N*=191)**		**Gram stain(*N*=32)**	
	**pos**	**neg**	**pos**	**neg**
**PCR**				
**Pos**	62	22	29	1
**Neg**	8	99	1	1
**Sensitivity**	73.81%		96.67	
**Specificity**	92.52 %		50	
**Accuracy**	AUC	0.832	0.733	
	95% CI	0. 768-0. 895	0.283-1	
	p	<0.001	0.028	
**Agreement**	*k*	0.675	0.467	
	95% CI	0.570-0.78	-0.160 - 1.000	
	p	<0.001	0.008	

*AUC* area under rock curve, *CI* confidence interval, *pos* positive, *neg* negative, *N* number of tested samples

## Discussion

Out of 191 observed carthorses, 84 (44%) were positive HCF nested PCR, which was higher than the clinical prevalence reported in various areas of the country. Overall EL prevalence were 26.2% in Debrezeit, Mojo and Nazareth; 21% in Bako and Ejaji towns; 18.8% in 28 town of Ethiopia, 24.9% in Woliso and 12% in Mekelle were observed [2, 4, 8, 16, 17].

Of the DNA isolated from buffy coat of the 70 presumably EL positive cases, 88.6% (62/70) were PCR positive which was significantly higher than the previous reported (63%; 17/27) on DNA isolated from whole blood samples of presumably EL positive animals [15]. Considering the intracellular nature of HCF particularly in neutrophils and macrophages, the buffy coat centrifugation could allow aggregation of the infected cells and probably the yeast fungi in the buffy coat layer, which could concentrate the HCF and increase the likelihood of PCR detection as indicated in diagnosis of visceral leishmaniasis [18]. Subclinical infection by HCF observed in 18.2% (22/121) of the apparently healthy carthorses. This is comparable with the findings in the highland horses of Ethiopia [15]. This proves the importance of PCR in the epidemiology of the EL, especially for screening purposes while moving horses from endemic to disease-free zones.

Considering the PCR result, adult carthorses were 9.66 times more likely to be infected by HCF than younger age group (AOR = 9.66, 95% CI 3.206–29.109, *p* < 0.001). This finding contradicts with previous reports of Ameni [4], where horses under six years of age were most susceptible. The high prevalence in adult aged carthorses could be due to the work related exposure to wounds and other abrasive materials that could potentially favor entry of the soil borne HCF [19]. Practically, horses above the age of four are engaged in fulltime work. Assembling of those adult horses in the carthorse stations during work favors the transmission of HCF by biting flies and the frequent use of similar bedding, harnessing, and saddlers for all owned carthorses favors rapid disease transmission [8, 20].

Carthorses that have poor body conditions were 1.299 and 9.523 times more likely to be positive for HCF using PCR than of those that have moderate and good body conditions, respectively (AOR = 0.105, 95% CI: 0.017–0.658, *p* = 0.016). This finding was consistent with the findings of other studies in central Ethiopia [4, 16, 20]. Carthorses with poor conditions tend to acquire EL because they are prone to wound infliction, which then attracts flies that increase the chance of infection.

The clinical features and microscopic appearances of the yeast forms of EL observed in the present study were in agreement with previous reports [2, 6, 8]. Cutaneous form of EL was the dominant case present [2, 21].

We evaluated the diagnostic accuracy of clinical observation in comparison with the nested PCR primer assay for diagnosis of HCF infection developed by [15]. Clinical signs used for diagnosis of EL showed a sensitivity of 73.81% and specificity 92.52%. The test agreement between PCR and clinical signs was good (*k* = 0.675, 95% CI 0.57–0.78). The lower sensitivity may be explained due to the presence of asymptomatic carriers and pre-clinical stages of the infection, in which the incubation period ranged from several weeks to six months [6]. These were positively identified using PCR, but had no symptoms to be detected using clinical observation. Yeast cells were present in 93.75% (30/32) of the stained smears, which was much higher than 52% reports of [15].

## Conclusions

The study indicated that EL is one of the major health threats of working horses. As compared to previous studies, PCR amplification from buffy coat DNA was more appropriate. Molecular prevalence of EL in both presumable EL cases and apparently healthy animals was high. The nested PCR could be potentially useful in disease control by regulatory authorities particularly in equine trade and prevention of unintentional introduction of HCF. The culture and isolation experiment indicated that isolation of HCF is possible, but external factors have to be controlled. We recommend that in settings with poor resources, where currently diagnosis of the disease is based on clinical signs, support by microscopic examination through gram stain smears from pus samples should be supplemented. However, early stages and potential carriers are undetected and remain at risk for the herd. Commercializing such techniques for routine use is far from the current practice. Therefore, the use of PCR as a screening test during movement or purchase is recommended. Moreover, sequencing and analysis of genetic phylogeny of the PCR positive samples shall be made to further understand the molecular epidemiology of HCF in the country.

## Methods

### Study animals and sampling

**Fig. 4:**
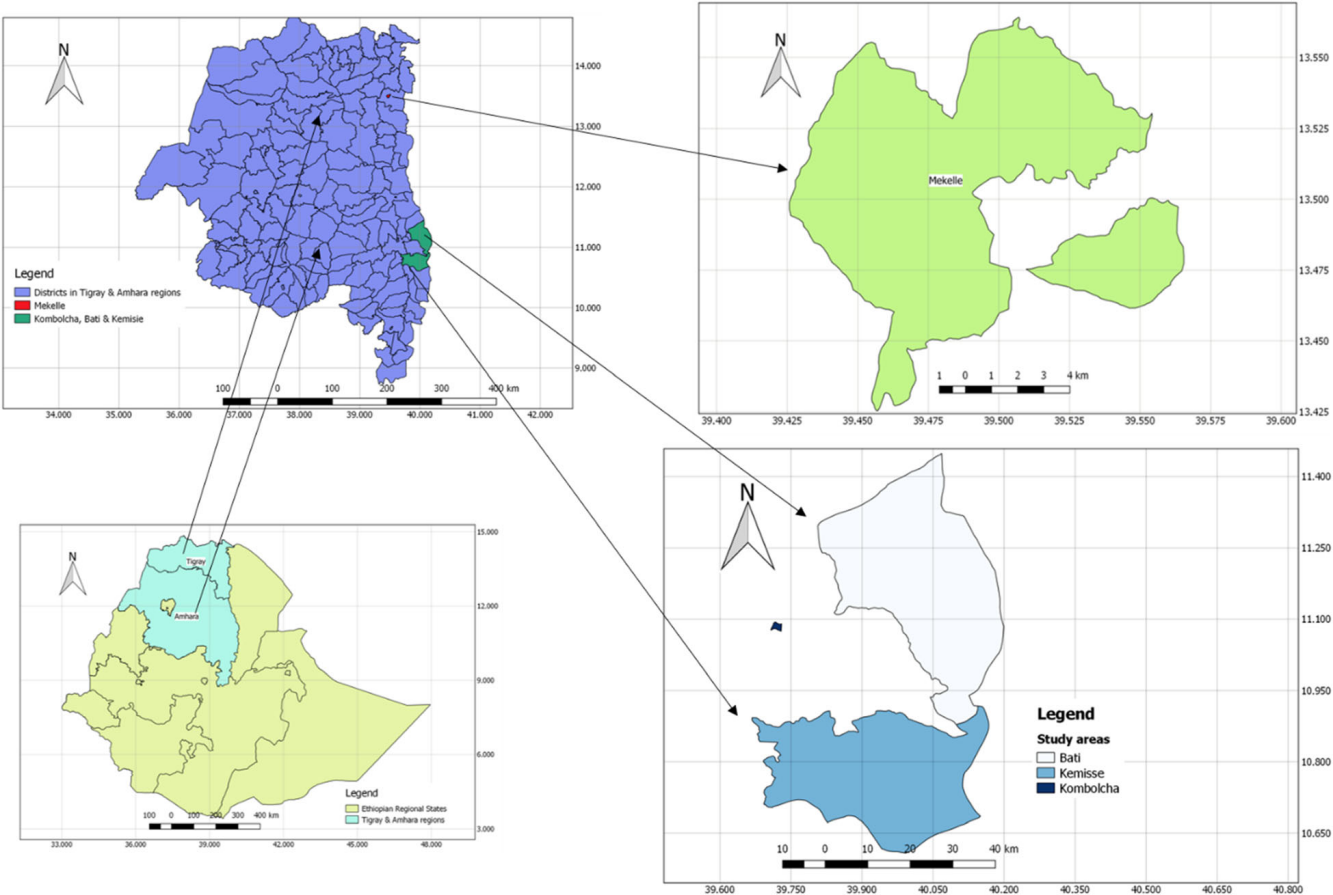
Map of study areas. Map produced by authors. The shape files for the country, study regions and districts were obtained from the Institute of Climate and Society of Mekelle University. Maps were produced using the applications of QGIS version 3.12.3 software

This cross-sectional study was conducted in Northern Ethiopia (Mekelle, Kombolcha, Bati, and Kamisse towns), where carthorses are used for the transportation of man and goods (Fig. 4). Carthorse owners were briefed about the purpose and relevance of the study. From willing owners, 191 carthorses with signs of lymphangitis or lymphadenitis or both (EL typical lesions) and apparently healthy ones were purposively selected [21]. The sample type and number of animals sampled from each study area is presented in Table 4.

**Table 4:** Sample type and number per each study area

Study town	Number of horses sampled	Sample type				
		**Carthorse with EL lesion**			**Apparently Healthy**	
		**buffy coat**		**pus**^a^	**buffy coat**	
Mekelle	61	36		25	25	
Kombolcha	52	6		1	46	
Bati	36	11		4	25	
Kamisse	42	17		2	25	
**Totals**	**191**	**70**		**32**	**121**	

^a^Pus samples for culture and isolation, and gram stain depends on the number of carthorses with un-ruptured epizootic lymphangitis (EL) lesions

From each study animal, records of body condition, age, and clinical condition were documented. Blood samples collected from the jugular veins, were centrifuged to separate the buffy coat for DNA extractions. Among the 191 carthorses, 32 of them with un-ruptured nodules of EL, pus samples were aseptically collected and dispensed into two universal bottles containing Sabarouds Dextrose Agar (SDA) containing chloramphenicol (0.5 g/ liter) and enriched with 2.5% glycerol and horse serum (each for isolation of yeast and mycelial forms of HCF). The remaining pus samples were smeared on glass slides for gram staining. Specimens were transported at 4℃ within a cool transport box to the College of Veterinary Sciences, Mekelle University, subsequently kept in -20℃ until use.

### Gram stain and culture

Pus smears fixed with methanol were stained with Gram’s stain for the identification of the yeast form of HCF microscopy (under 1000x magnification) [6]. The SDA culture media inoculated with pus was incubated at either 26 ℃ or 37℃ with 5% CO2, respectively to isolate mycelial and yeast form [21–23]. The culture was checked periodically, and Gram-stained preparations were made from suspicious growth [2].

### PCR

DNA was extracted from buffy coat samples and yeast culture according to the recommended procedures indicated in Qiagen DNeasy blood and Tissue Kit (Lot 157,043,215, QIAGEN, Hilden, Germany). Primers were synthesized in the Genetic Facility of Iowa State University, Ames, Iowa, USA. HCF positive control DNA obtained from Prof. Dr. Gobena Ameni, Aklilu Lema Institute of Pathobiology, Addis Ababa University. Except a little modification in the cycling conditions, primers and nested PCR protocol were adopted from [15]. In a 25 µl reaction volume, 0.25 µl of DNA polymerase, 0.3 µM of each primer and 2.5 µl of template DNA were used. The first cycling condition was 95 °C for 10 min and 39 cycles of 94 °C for 1 min, 49 °C for 1 min, and 72 °C for 1 min followed by a final extension cycle of 72 °C for 10 min. Cycling condition of the second round was the same as that of the first round except the annealing temperature was raised to 55 °C for 1 min. The expected product was 514 bp and was visualized via electrophoresis at 92 V for 45 min on a 2% (wt/vol) agarose gel stained with Ethidium bromide [15].

### Data processing and analysis

Generated data entered into Microsoft Excel, imported and analyzed using Statistical Package for Social Sciences (SPSS) software version 21.0. Descriptive statistics and categorical results presented using Tables. Associations between exposure and outcome variables were assessed using binary logistic regression analysis. Binomial logistic regression through crude odds ratio (COR) was used to asses strength of association of EL with body condition score, age and study area. For variables that showed significant difference through COR, to avoid confounding factors, multiple logistic regression through adjusted odds ratio (AOR) was computed. Test agreement between two diagnostic tests was computed by Kappa test statistics. The level of agreement between the diagnostic tests determined using Cohen’s kappa coefficient [24, 25]. Probability (*p*) values < 0.05 were considered as significant.

## Data Availability

The datasets used and/or analyzed during the current study are available from the corresponding author on reasonable request.

## Abbreviations

COR: Crude odds ratio
DNA: Deoxyribonucleic acid
EDTA: Ethylene diamine tetra acetic acid
EL: Epizootic lymphangitis
*HCC*: *Histoplasma capsulatum var. capsulatum*
HCF: *Histoplasma capsulatum var. faciminosum*
Kms: Kilometers
masl: Meter above sea level
mm: Millimeter
PCR: Polymerase chain reaction
rpm: Revolution per minute
US$: United States Dollar
